# Meta-Milgram: An Empirical Synthesis of the Obedience Experiments

**DOI:** 10.1371/journal.pone.0093927

**Published:** 2014-04-04

**Authors:** Nick Haslam, Steve Loughnan, Gina Perry

**Affiliations:** School of Psychological Sciences, University of Melbourne, Parkville, Victoria, Australia; University of Vienna, Austria

## Abstract

Milgram's famous experiment contained 23 small-sample conditions that elicited striking variations in obedient responding. A synthesis of these diverse conditions could clarify the factors that influence obedience in the Milgram paradigm. We assembled data from the 21 conditions (*N* = 740) in which obedience involved progression to maximum voltage (overall rate 43.6%) and coded these conditions on 14 properties pertaining to the learner, the teacher, the experimenter, the learner-teacher relation, the experimenter-teacher relation, and the experimental setting. Logistic regression analysis indicated that eight factors influenced the likelihood that teachers continued to the 450 volt shock: the experimenter's directiveness, legitimacy, and consistency; group pressure on the teacher to disobey; the indirectness, proximity, and intimacy of the relation between teacher and learner; and the distance between the teacher and the experimenter. Implications are discussed.

## Introduction

The Milgram study is arguably the most iconic experiment in the history of psychology. In the fifty years since it was conducted, debate about its implications has spread far beyond the academic literature of social psychology and into the culture at large. Scholars continue to discuss whether Milgram demonstrated the capacity for evil in everyday people, the roots of the Holocaust, or the ethical limitations of psychological research. Arguments continue on the nature of authority and the meaning of obedience within Milgram's paradigm [1] and how the study's findings should be theorized [2]. Attempts have been made to replicate it with mixed results [3], [4] and the original data have been re-examined [5]. Meanwhile, archival scholarship continues to examine the origins of Milgram's work [6] and to unearth troubling discrepancies between its public representation and how its methodology was executed in practice [7].

The most famous of Milgram's findings is associated with the best-known version of his experiment. A substantial majority of study participants, recruited from the general public as “teachers” in a study of paired associates learning, continued to shock an unresponsive and possibly dying “learner” up to the maximum 450 volts at the behest of the “experimenter.” (Although it remains unclear and somewhat controversial how this behavior should be conceptualized, and even whether it is best described as ‘obedience’ [7], we use that term as shorthand to describe the progression of experimental subjects to 450 volts.) This rate (62.5%) exceeded by a factor of 500 the figure estimated by psychiatrists who read the study protocol [8]. It is the shock value of this finding – the fact that a majority of ordinary people were apparently capable of destructive obedience – that has triggered the enduring interest in Milgram's work, and the desire to make sense of it.

**Figure 1:**
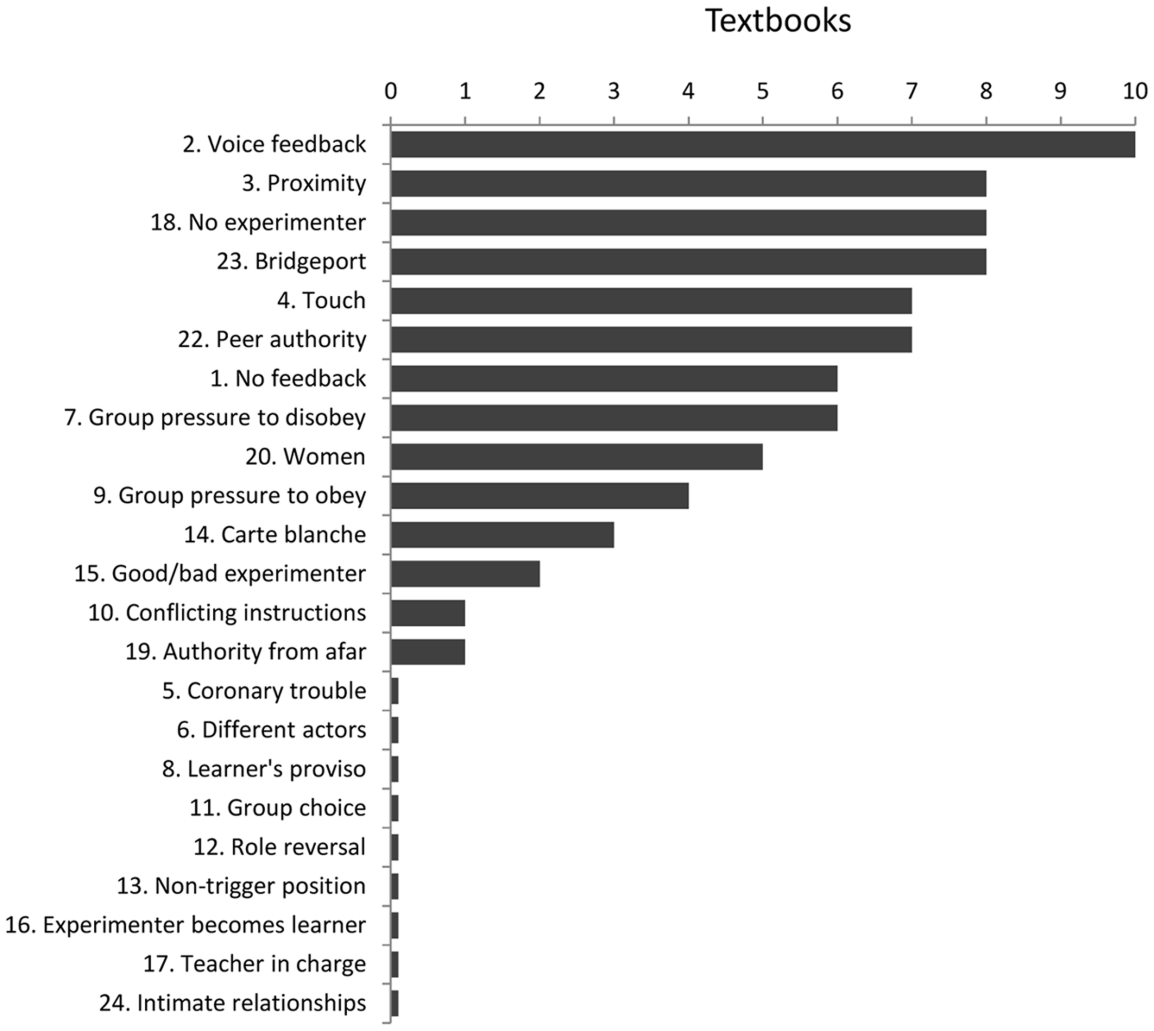
Number of social psychology textbooks (*N* = 10) referring to the 23 experimental conditions.

Less well-known is the fact that this finding represents just one of 23 diverse experimental conditions that Milgram conducted, which varied enormously in levels of obedient responding. Only 18 of these were reported in the monograph that reported the study [8]. The full set of 23 conditions, numbered in the order they were carried out from August 1961 to May 1962 and in accordance with Milgram's notes from the Yale University archive, are sketched in Table 1. Although several conditions are familiar to many psychologists, others are obscure and rarely discussed. For example, a survey of ten social psychology textbooks [9], [10], [11], [12], [13], [14], [15], [16], [17], [18] shows that although the average text refers to 7.6 conditions, nine conditions go completely unmentioned (see Figure 1, which lists conditions according to Milgram's numbering: see Table 1).

**Table 1:** Summary of study conditions (E =  experimenter, L =  learner, T =  teacher).

No.	Name	Brief description
1	No feedback	Like baseline condition (2) but L does not cry out
2	Voice feedback	Baseline condition with 1 T in separate room from L, with 1 E present
3	Proximity	Like baseline condition but with T in same room as L, seated behind him
4	Touch	Like baseline condition but with T holding L's hand to the shock plate
5	Coronary trouble	Like baseline but L mentions heart trouble at beginning of the experiment and protests about it later
6	Different actors	Identical to condition 5 but with a different actors playing Learner and Experimenter
7	Group pressure to disobey	Like baseline condition but with 3 Ts: two (confederates) defy the E, who urges the participant T to continue shocks
8	Learner's proviso	Like baseline condition but at study outset L insists that he will only agree to take part if he can leave when he wants
9	Group pressure to obey	Like condition 7 but the 2 confederate Ts pressure the participant T to obey the E's directions
10	Conflicting instructions	Like baseline condition but E urges T to stop the shocks and L urges him to continue (*obedience means not going to 450V*)
11	Group choice	Like condition 7 but Ts can determine shock level (lowest of their 3 bids): confederate Ts go first and always increase
12	Role reversal	Like baseline condition but E and L swap roles (*obedience means not going to 450V*)
13	Non-trigger position	Like condition 7 but participant T reads word pairs while one of the confederate Ts administers shocks
14	Carte blanche	Like baseline condition but T decides the level of shocks on his own, without E's directions
15	Good/bad experimenter	Like baseline condition but there are 2 Es who give conflicting directions: one to stop, one to continue
16	Experimenter becomes learner	Like baseline condition but with 2 Es, one of whom volunteers to serve as L when original L is said to be unavailable
17	Teacher in charge	Like baseline condition but with 2 Ts, one of whom (a confederate) is given authority to choose shock levels when E is called away
18	No experimenter	Like baseline condition but E is called away and tells T to continue the experiment on his own, leaving E's phone number
19	Authority from afar	Like condition 18 but E leaves pre-recorded instructions for T to follow
20	Women	Like baseline condition but all Ts are female
21	Expert judgment	Psychiatrists and laypeople read the baseline study protocol and estimate level of obedience (*not a true empirical condition*)
22	Peer authority	Like condition 17 but confederate T suggests shock levels without being given authority to chose them and E leaves them to T's discretion
23	Bridgeport	Like condition 5 but study conducted in dingy Bridgeport office rather than at Yale
24	Intimate relationships	Like baseline condition but the L is a friend or relative of the T

An analysis of the data from the 23 study conditions could establish which of the situational properties that vary across conditions covary with participants' rates of progression to maximum voltage. However, this task is made difficult by the ad hoc nature of the conditions [6], which compose a patchwork of methodological elements rather than a systematic investigation of well-articulated experimental factors. Milgram often designed new conditions to explore specific situational factors that might influence obedience, such as the well-known Bridgeport replication, which repeated the original Yale study in an industrial setting. These specific variations are commonly reported as pairwise comparisons of study conditions, each of which had a small sample size (usually 40, but sometimes only 20). Thus the 47.5% obedience rate in Bridgeport is usually contrasted with the 62.5% rate for the comparable condition at Yale, and interpreted as evidence that the status, legitimacy, or prestige of the setting influences obedience. As a result, it is difficult to offer any definitive conclusions about Milgram's findings based on anything more than piecemeal analysis of small sample variations within the larger experimental program.

A better way to examine the experimental factors that influence obedience in Milgram's research would be to synthesize its findings by amalgamating his conditions in a manner akin to meta-analysis and assessing moderators of obedience in the combined sample. The combined sample of the 23 conditions is a substantial 780 participants. No analysis that synthesizes conditions from Milgram's study to examine determinants of obedience has previously been conducted. Packer [5] carried out a meta-analysis of eight conditions but focused on the critical voltage levels at which disobedient participants refused to continue rather than on differences in levels of obedience across conditions. Reicher, Haslam, and Smith [19] correlated levels of obedience in 15 of the 23 conditions with ratings by social psychologists and students of the teacher's probable level of identification with experimenter and learner, but did not examine characteristics internal to the Milgram study as predictors of obedience levels.

Deciding how to systematically characterize the variations among Milgram's conditions in a way that might illuminate differences in obedience rates is no easy task. Milgram himself did not provide a systematic classification of his conditions beyond simply clustering them into those exploring the “immediacy of the victim”, “presence of an authority figure”, and “group experiments”. Other writers have identified numerous differentiating characteristics, often labeled in multiple ways. Sometimes these characteristics have been integrated into two broad components: those that connect the teacher to the experimenter and those that link the teacher to the learner. Gilovich et al. [12] refer to these sets of features as “tuning out [or in] the experimenter” and “tuning in [or out] the learner”. Other writers offer alternative distinctions. For example, Aronson et al. [9] distinguish informational and normative influences. Myers [15] proposes that the primary factors are the victim's distance, the authority's closeness and legitimacy, institutional authority, and the liberating effect of disobedient peers. Sutton and Douglas [17] sort the relevant factors into proximity of experimenter to teacher, proximity of learner to teacher, authority of the situation, authority or status of the experimenter, and group pressure.

**Figure 2:**
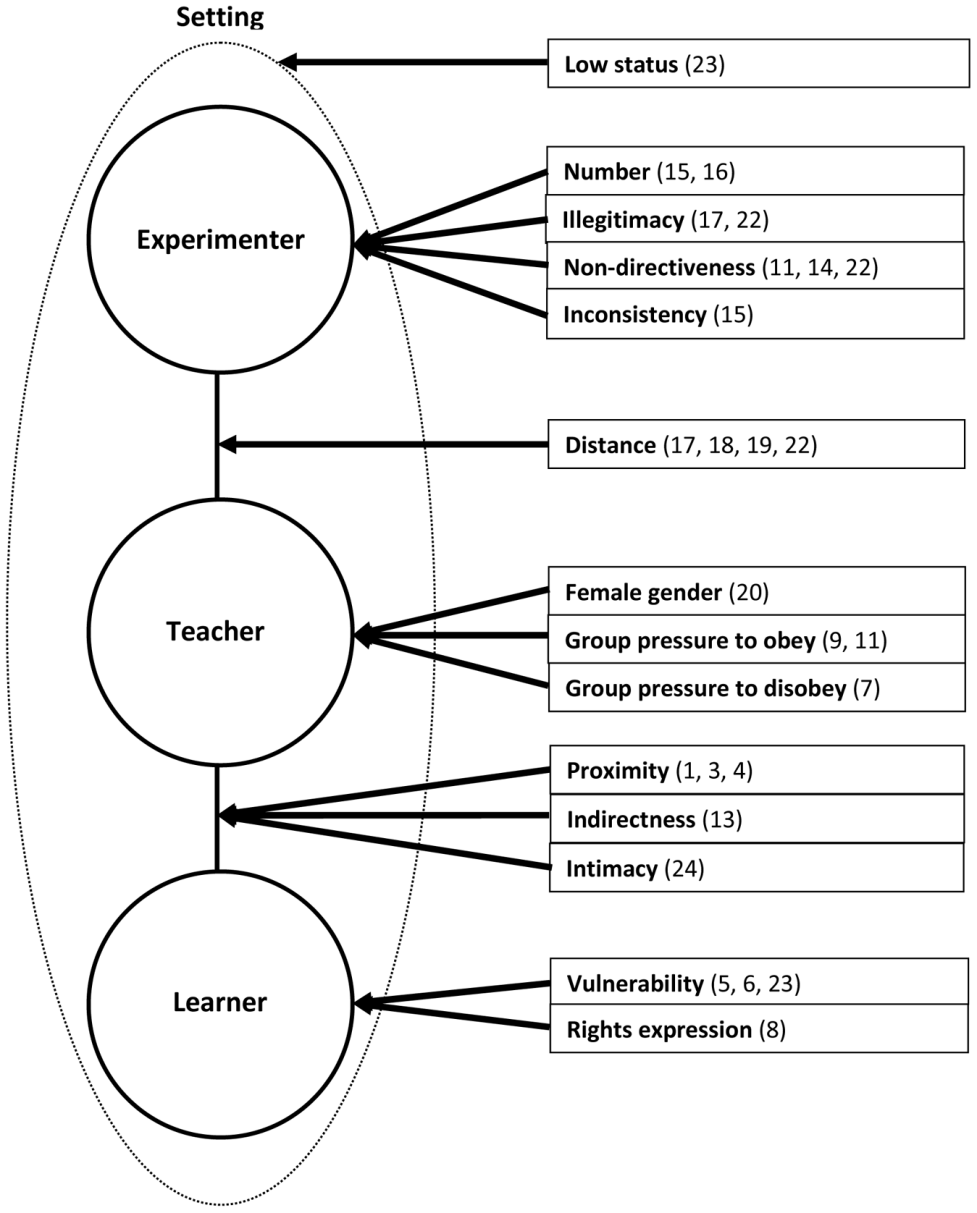
Schematic of coding factors (relevant conditions in parentheses).

Rather than begin with a particular classification of factors that might influence obedience levels across the study conditions, we began with an abstract schema of Milgram's experiment and attempted to fit his experimental variations into this schema. By this means we attempted to determine inductively which of a large set of experimental features are independently associated with variations in obedience. Our schema (see Figure 2) started from the recognition that the Milgram experiment involves three hierarchically organized roles (Experimenter, Teacher, Learner) and two relationships between them (Experimenter-Teacher and Teacher-Learner), there being no unmediated relationship between Experimenter and Learner. By “relationship” we mean any intrinsically relational aspect of their connection, such as distance or intimacy. With one exception the factors that Milgram varied across his conditions can be located within one of the three roles or the two relationships. The exception is the setting in which the experiment was conducted (i.e., Yale versus Bridgeport). The schema therefore identifies six classes of factors that Milgram manipulated across his study conditions.

Having developed a reasonably comprehensive set of study properties to capture the variations among Milgram's conditions, we conducted a statistical analysis to determine which of these factors were independently associated with obedience levels. Treating Milgram's conditions as a single study with a large sample, rather than as a variegated collection of studies with small samples, allows a powerful test of the situational influences on obedience within his paradigm. The aim of our study was to determine which of the many potential influences were statistically reliable, rather than to test a particular theory of obedience or interpretation of the Milgram study. Nevertheless, any such theory or interpretation must be consistent with the determinants that are found to be efficacious.

## Materials and Methods

### Ethics statement

This report presents a re-analysis of publically available, previously published data originally collected by Milgram and his colleagues in 1961 and 1962, prior to the advent of institutional review boards. No informed consent was required at that time by Yale University. Participants provided uninformed verbal consent and signed a waiver absolving Yale University of legal responsibility.

### Selection of conditions

Milgram's study included 23 conditions in which participants completed a variation of the obedience protocol. Another variation, sometimes referred to as condition 21, assessed levels of obedience predicted by laypeople and psychiatrists rather than actual behavior, and is therefore not an experiment. Two conditions – numbers 10 (“conflicting instructions”) and 12 (“role reversal”) – differ from the others in that proceeding to the 450 V shock involves *dis*obeying the experimenter, and because of this fundamental difference in the meaning of the dependent measure these conditions were excluded from the analysis. The analysis therefore included 21 of the 23 conditions, and 740 of the 780 (94.9%) total participants.

Four conditions with complex, two-part designs allow two alternative ways of counting the number of obedient participants. Obedience levels from part B of condition 15 (“good experimenter, bad experimenter”) were selected because part A ended at 150 V and therefore did not allow all participants the opportunity to defy the experimenter. Parts A of conditions 17 (“teacher in charge”), 18 (“no experimenter”), and 22 (“peer authority”) were selected because they all allowed participants to proceed all the way to 450 V before part B was initiated.

### Coding

To determine which variations among study conditions were independently associated with differences in obedience rates, we developed a set of codes to distinguish the conditions. Development of the codes was guided by two considerations: codes should identify distinctions recognized by Milgram or other scholars, and they should be reasonably exhaustive, ideally yielding a unique configuration of codes for each condition. The latter goal was successfully met with two exceptions. Conditions 5 and 6 (“coronary trouble” and “different actors”) were coded identically because they differed only in the actors playing the learner and experimenter roles. Conditions 18 and 19 (“no experimenter” and “authority for afar”) were coded identically because in both conditions the experimenter departs after explaining the study and leaves a phone number on which he can be contacted, with no other significant procedural differences.

A total of 14 codes were developed and organized into our six-part schema (see Figure 2). Some codes pertained to variations in properties of the three roles in the study: the learner, the teacher, and the experimenter. Others pertained to the relations between pairs of protagonists or roles: the teacher-learner relation and the experimenter-teacher relation. Finally, one code related to the overall setting or context of the study. With one exception, all codes were dichotomous with “0” representing the more common default position and “1” representing the deviant condition, which guided the naming of the coded properties. The codes are described according to the six-part schema below, and are summarized in Tables 2 and 3, along with their associated obedience rates.

**Table 2:** Summary of conditions including codes related to the learner, teacher, and teacher-learner relation.

				Learner properties		Teacher properties			Teacher-learner properties		
No.	Condition label	*N*	*N* “obey”	Vulnerability	Rights expression	Female gender	Group pressure to obey	Group pressure to disobey	Intimacy	Proximity	Indirectness
1	No feedback	40	26	0	0	0	0	0	0	0	0
2	Voice feedback	40	25	0	0	0	0	0	0	1	0
3	Proximity	40	16	0	0	0	0	0	0	2	0
4	Touch	40	12	0	0	0	0	0	0	3	0
5	Coronary trouble	40	26	1	0	0	0	0	0	1	0
6	Different actors	40	20	1	0	0	0	0	0	1	0
7	Group pressure to disobey	40	4	0	0	0	0	1	0	1	0
8	The learner's proviso	40	16	0	1	0	0	0	0	1	0
9	Group pressure to obey	40	29	0	0	0	1	0	0	1	0
10	Conflicting instructions	20	20	Not included in analysis							
11	Group choice	40	7	0	0	0	1	0	0	1	0
12	Role reversal	20	20	Not included in analysis							
13	Non-trigger position	40	37	0	0	0	0	0	0	1	1
14	Carte blanche	40	1	0	0	0	0	0	0	1	0
15	Good/bad experimenter	20	4	0	0	0	0	0	0	1	0
16	Experimenter → learner	20	13	0	0	0	0	0	0	1	0
17	Teacher in charge	20	11	0	0	0	0	0	0	1	0
18	No experimenter	40	9	0	0	0	0	0	0	1	0
19	Authority from afar	40	15	0	0	0	0	0	0	1	0
20	Women	40	26	0	0	1	0	0	0	1	0
22	Peer authority	20	4	0	0	0	0	0	0	1	0
23	Bridgeport	40	19	1	0	0	0	0	0	1	0
24	Intimate relationships	20	3	0	0	0	0	0	1	1	0

**Table 3:** Summary of conditions including codes related to the experimenter, the experimenter-teacher relation, and the setting.

				Experimenter properties				Experimenter-teacher properties	Setting property
No.	Condition label	*N*	*N* “obey”	Number	Illegitimacy	Non-directiveness	Inconsistency	Distance	Low status
1	No feedback	40	26	0	0	0	0	0	0
2	Voice feedback	40	25	0	0	0	0	0	0
3	Proximity	40	16	0	0	0	0	0	0
4	Touch	40	12	0	0	0	0	0	0
5	Coronary trouble	40	26	0	0	0	0	0	0
6	Different actors	40	20	0	0	0	0	0	0
7	Group pressure to disobey	40	4	0	0	0	0	0	0
8	The learner's proviso	40	16	0	0	0	0	0	0
9	Group pressure to obey	40	29	0	0	0	0	0	0
10	Conflicting instructions	20	20	Not included in analysis					
11	Group choice	40	7	0	0	1	0	0	0
12	Role reversal	20	20	Not included in analysis					
13	Non-trigger position	40	37	0	0	0	0	0	0
14	Carte blanche	40	1	0	0	1	0	0	0
15	Good/bad experimenter	20	4	1	0	0	1	0	0
16	Experimenter → learner	20	13	1	0	0	0	0	0
17	Teacher in charge	20	11	0	1	0	0	1	0
18	No experimenter	40	9	0	0	0	0	1	0
19	Authority from afar	40	15	0	0	0	0	1	0
20	Women	40	26	0	0	0	0	0	0
22	Peer authority	20	4	0	1	1	0	1	0
23	Bridgeport	40	19	0	0	0	0	0	1
24	Intimate relationships	20	3	0	0	0	0	0	0

#### Learner properties

Two codes referred to properties of the learner. “*Vulnerability*” refers to three conditions (5 [“coronary trouble”], 6 [“different actors”] & 23 [“Bridgeport”]) in which the learner mentions heart trouble at the beginning of the experiment, augmenting the heart-related concerns that are part of the standard script in the other conditions. Thus conditions 5, 6, and 23 were coded “1” and all other conditions coded “0”. “*Rights expression*” refers specifically to condition 8 (“learner's proviso”), where at the outset the learner says he will only participate if he is able to leave when he wants. Condition 8 was therefore coded “1” and all others “0”.

#### Teacher properties

Three codes referred to properties of the teacher role. “*Female gender*” pertains to the single condition (20 [“women”]) that employed female participants, so this condition was coded “1” and all others “0”. “*Group pressure to obey*” refers to the distinction between two conditions (9 [“group pressure to obey”] & 11 [“group choice”]) in which multiple teachers (actually confederates) exert pressure on the participant teacher to escalate the shocks (coded “1”) and all other conditions (coded “0”), where no such pressure was exerted. “*Group pressure to disobey*” contrasted one condition (7 [“group pressure to disobey”]) involving pressure within the teacher group against obeying (coded “1”) and all other conditions (coded “0”). These group pressure variants are discussed in terms of “normative influence,” “social consensus”, or “social support” by some writers on the Milgram study.

#### Experimenter properties

Four experimenter properties were coded. “*Number*” distinguishes two conditions (15 [“good experimenter, bad experimenter”] & 16 [“experimenter becomes learner”]) employing two experimenters, both coded “1”, from all others, coded “0”. (Condition 18, entitled “no experimenter,” actually has an experimenter who meets the participant before being called away.) “*Illegitimacy*” – referred to as low experimenter “status” or “authority” by some writers – distinguishes two conditions (17 [“teacher in charge”] & 22 [“peer authority”], both coded “1”) in which an apparent participant (actually a confederate) takes over the experimenter role, from all other conditions, coded “0”, where the experimenter is identified as a scientist or researcher. “*Non-directiveness*” distinguishes three conditions (11 [“group choice”], 14 [“carte blanche”] & 22 [“peer authority”], all coded “1”) in which no explicit direction is given to increase the shocks (shock level is instead left to the discretion of the participants) from all other conditions, where such a direction is always given (coded “0”). Finally, “*Inconsistency*” separates one condition (15 [“good experimenter, bad experimenter”]) in which the experimenter role is internally conflicted (coded “1”) from all other conditions (coded “0”), where the role is consistent, most often because there is a single, unwavering experimenter.

#### Teacher-learner relation properties

Three properties of the relationship between teacher and learner were coded. “*Intimacy*” distinguishes the little-known condition 24 (“intimate relationships”), in which the learner was a friend or relative of the teacher (coded “1”), from all other conditions (coded “0”), where the two were strangers. “*Proximity*” – sometimes referred to as “immediacy” – captures degrees of distance between teacher and learner. Least proximal is condition 1 (“no feedback”, coded “0”), where the learner is in an adjoining room and does not cry out, followed by the baseline condition 2 (“voice feedback”, coded “1”) in which the learner is in an adjoining room but screams. Condition 3 (“proximity”, coded “2”) has the learner seated close behind the teacher in the same room, and condition 4 (“touch”, coded “3”) has the teacher holding the learner's hand to the shock-plate. All other conditions, which followed the baseline condition in this regard, were coded “1”. Finally, the “*Indirectness*” code distinguished condition 13 (“non-trigger position”, coded “1”), where the participant is a teacher who reads the word pairs while another administers the shocks, from all other conditions (coded “0”), where the teacher's role in shocking the learner was unmediated.

#### Experimenter-teacher relation properties

One code, “*Distance*”, captured variation among conditions in the relation between experimenter and teacher. Four conditions in which the experimenter absents himself during the study (17 [“teacher in charge”], 18 [“no experimenter”], 19 [“authority from afar”] and 22 [“peer authority”]) (coded “1”), are distinguished from all other conditions (coded “0”), where the experimenter is physically present in the experimental situation throughout.

#### Setting property

A final code pertained to the setting or context of the experiment, distinguishing condition 23 (“Bridgeport”), conducted in an industrial neighborhood (coded “1”), from all other conditions (coded “0”), which were carried out on Yale University's ivied campus. The code was called “*Low status*”, but other writers have referred to it as low “prestige”, “legitimacy”, “institutional authority”, or “authority of the situation.”

All coding was based on published descriptions of the conditions and on Milgram's original notes, accessed by the third author at the Yale University archives. The original, hand-written data summary sheets were also used to confirm obedience rates for each condition. *Data file construction*.

A data file (*N* = 740) was reconstructed using the known sample sizes for each condition (*n* = 40 for 16 conditions, *n* = 20 for 5 conditions) and the number of participants in each condition who proceeded to deliver the 450 V shock. Obedience was coded dichotomously as delivering this highest shock, consistent with standard practice and in recognition of the marked irregularity of the distribution of highest voltages delivered, which renders continuously scored voltage level statistically problematic as a dependent measure.

## Results

Across the 21 conditions the proportion of obedient participants was 323/740 (43.6%). Table 4 presents rates of obedience as a function of each dichotomous code. Eight codes were associated with differential rates of obedience. Obedience rates were higher for more vulnerable learners (*p* = .011), for female teachers (*p* = .005), and for more indirect teacher-learner relations (*p*<.001). Rates were lower when there was more group pressure for experimenters to disobey (*p*<.001), when the teacher-learner relation was more intimate (*p* = .009), when the experimenter was non-directive (*p*<.001) and inconsistent (*p* = .031), and when the experimenter-teacher relation was more distant (*p* = .007). A comparable test of the bivariate relationship between obedience and the one non-dichotomous code, “Proximity”, showed that greater proximity between teacher and learner was associated with lesser obedience (Spearman *r* = −.37, *p*<.001).

**Table 4:** Proportion of obedient participants as a function of code value.

Code	Coded 1	Coded 0	χ^2^_(1)_	*p*
**Experimenter (E)**				
Number	0.43	0.44	0.02	.879
Illegitimacy	0.38	0.44	0.65	.420
Non-directiveness	0.12	0.49	47.09	<.001
Inconsistency	0.20	0.44	4.67	.031
**Teacher (T)**				
Female gender	0.65	0.42	7.84	.005
Group pressure to obey	0.45	0.43	0.07	.796
Group pressure to disobey	0.10	0.46	19.47	<.001
**Learner (L)**				
Vulnerability	0.54	0.42	6.44	.011
Rights expression	0.41	0.44	0.23	.632
**Experimenter-Teacher relation (E-T)**				
Distance	0.33	0.46	7.24	.007
**Teacher-Learner relation (T-L)**				
Intimacy	0.15	0.44	6.86	.009
Indirectness	0.93	0.41	41.03	<.001
**Setting**				
Low status	0.48	0.43	0.26	.614

In view of the redundancy among the predictor codes, a logistic regression analysis was conducted to determine which condition properties were independently associated with obedience levels. “Proximity,” was coded in increasing order of closeness from 0 to 3. Although linear, quadratic, and cubic effects for this variable were estimated within the model, only the linear effect was of interest. The model accounted for substantial variation in obedience (Nagelkerke *R* ^2^ = 0.30, *p*<.01) and eight of the 14 coded variables independently predicted this outcome. Findings of the analysis are summarized in Table 5, where positive values of *B* signify that conditions higher in the property named by the code tend to have higher rates of obedience, and negative values signify the reverse.

**Table 5:** Summary of logistic regression analysis.

Code	*B*(SE)	Wald	d.f.	*p*
**Experimenter (E)**				
Number	0.32 (0.55)	0.34	1	.560
Illegitimacy	1.37 (0.47)	8.50	1	.004
Non-directiveness	−2.79 (0.39)	50.45	1	<.001
Inconsistency	−2.01 (0.73)	7.56	1	.006
**Teacher (T)**				
Female gender	0.32 (0.44)	0.53	1	.467
Group pressure to obey	0.78 (0.40)	3.77	1	.052
Group pressure to disobey	−2.49 (0.60)	17.04	1	<.001
**Learner (L)**				
Vulnerability	0.06 (0.37)	0.00	1	.987
Rights expression	−0.70 (0.44)	2.57	1	.109
**Experimenter-Teacher relation (E-T)**				
Distance	−1.14 (0.38)	8.92	1	.003
**Teacher-Learner relation (T-L)**				
Intimacy	−2.03 (0.69)	8.61	1	.003
Indirectness	2.22 (0.67)	10.98	1	.001
Proximity		12.00	3	.007
(linear)	−1.14 (0.34)	11.55	1	.001
(quadratic)	−0.59 (0.32)	0.03	1	.855
(cubic)	0.14 (0.31)	0.21	1	.648
**Setting**				
Low status	−0.40 (0.39)	1.07	1	.614

Table 5 indicates that three of the four Experimenter variables were associated with obedience. Higher obedience resulted when experimenters gave authoritative directions rather than leaving shock levels to teachers (*p*<.001), and lower obedience occurred when their directions were inconsistent (i.e., differing between experimenters: *p* = .006). Surprisingly, obedience rates were somewhat higher when the authority was illegitimate (i.e., a peer rather than a researcher: *p* = .004), an effect that might reflect collinearity among predictors given the lack of bivariate association between illegitimacy and obedience shown in Table 4. The presence of multiple experimenters did not influence obedience levels (*p* = .56).

Similarly mixed findings were obtained for the three Teacher variables, only one of which had a significant effect. Pressure to disobey from a group of teachers substantially decreased obedience (*p*<.001). However, pressure to obey from a group of teachers only marginally increased it (*p* = .052) and teacher gender had no effect (*p* = .467), the higher rate of obedience obtained for female teachers in the bivariate analysis disappearing when other variables were statistically controlled. Neither of the two Learner variables – vulnerability (*p* = .987) or rights expression (*p* = .109) – had significant effects on obedience, the bivariate vulnerability association also disappearing when other variables were held constant.

Turning to the relationship and setting variables, distance between the Experimenter and Teacher had an effect (*p* = .003), such that greater distance between them was associated with lesser obedience. All three Teacher-Learner relation variables had significant effects: conditions in which the teacher and learner were more proximal (*p* = .001), more intimate (*p* = .003), and more directly related (*p* = .001) had lower rates of obedient responding. Finally, the Setting variable, “low status”, was unrelated to obedience (*p* = .301).

Although the six code groupings – learner, teacher, experimenter, teacher-learner relation, experimenter-teacher relation, and setting properties – contain different numbers of codes, the relative magnitude of their effects offers some insight into the importance of these property types within the set of conditions that Milgram employed. Table 6 presents Nagelkerke *R* ^2^ values for each set of codes, which suggest that three property types - Experimenter, Teacher-Learner relation, and Teacher - are pre-eminent determinants of obedience rates across Milgram's 21 study conditions.

**Table 6:** Relative predictive contribution of the six code sets.

Code set	Variables	Nagelkerke *R* ^2^
Experimenter (E)	4	0.116
Experimenter-Teacher relation (E-T)	1	0.013
Teacher (T)	3	0.052
Teacher-Learner relation (T-L)	3	0.110
Learner (L)	2	0.012
Setting	1	<0.001

## Discussion

Our analysis indicates that many properties of Milgram's study conditions were associated with rates of obedient responding. These eight properties are diverse, pertaining to aspects of two of the three roles in the study – Teacher and Experimenter – as well as to both of the relationships between roles: Teacher-Experimenter and Teacher-Learner. Although our study brackets off the issue of how obedience within the Milgram study should be understood and takes no theoretical position on that issue, the number and diversity of these properties present a challenge for any encompassing account of obedience in the Milgram paradigm.

The significant predictors of obedience in our analysis are clearly disparate. The most powerful effects, in decreasing order, are the Experimenter's non-directiveness, the Teachers' group pressure to disobey, the Teacher-Learner relation's proximity and indirectness, the Teacher-Experimenter relation's distance, the Teacher-Learner relation's intimacy, and the Experimenter's illegitimacy and inconsistency. Several of these effects are well-established within the literature on the Milgram study, such as proximity, group pressure to disobey, and distance between Experimenter and Teacher. Others have been largely overlooked.

For example, few of the textbooks whose coverage was sampled in Figure 1 recognized the importance of the Experimenter's directiveness vs. non-directiveness, failing to note the very low levels of obedience in the “Carte blanche” and “Group choice” conditions. Proceeding to the 450 V shock rarely occurs if the authority figure does not give explicit commands to escalate the shocks, even if pressure to escalate is coming from fellow teachers (i.e., in the “Group choice” condition). Few textbooks noted the role of inconsistency among Experimenters in reducing obedience, neglecting to cite the “Good experimenter/bad experimenter” condition, where a benign experimenter almost completely overrode the power of the standard “bad” experimenter to induce compliance. No textbooks in our sample recognized the role of the indirectness of the relation between Teacher and Learner, failing to mention the “Non-trigger position” condition and its very high rates of obedience. Similarly, no textbooks acknowledged how the intimacy of the relationship between Teacher and Learner reduces obedience. Participants shocked learners with whom they had an existing social bond at less than one quarter the rate as when the learners were strangers. These four factors deserve greater attention in commentaries on Milgram's work.

Just as some factors that significantly predict obedience have been overlooked, other well-publicized factors were not significant predictors in our analysis or had unexpected effects. In particular, the analysis of textbook coverage shows that Milgram's replication of his study in Bridgeport, and his examination of the role of experimenter legitimacy through the “Peer authority” condition, attract substantial attention. However, the status of the setting was not associated with obedience in our systematic analysis of the 21 conditions, with levels similar regardless of the prestige of the experimental situation. Moreover, the illegitimacy of the authority was associated with *higher* obedience levels. Although this finding may be unreliable, it clearly contradicts the expectation that more legitimate authorities generate greater obedience in the Milgram paradigm. Although obedience was low (20%) in the “Peer authority” condition, our analysis suggests that this was probably due to the non-directive instruction in that condition rather than to the illegitimacy of the person proposing the shock levels (i.e., a peer rather than an identified researcher). In “Teacher in charge”, another condition where a peer was drafted into the authority role, obedience rates were a relatively high 55%, challenging the standard interpretation that peers, as illegitimate authorities, are not obeyed. In short, the importance of the prestige of the situation and the legitimacy of the authority may have been over-estimated in past interpretations of Milgram's work.

Such interpretations have often distinguished two components of the experimental situation. On the one hand, the Experimenter exerts a more or less authoritative influence on the Teacher, and on the other, the Learner generates more or less compassion or moral concern in that Teacher. The relative strength of these two influences is taken to determine rates of obedience, whether it is understood in terms of the Teacher's relative identification with Experimenter and Learner [19] or “tuning them in (or out)” [15]. Milgram's conditions cannot definitively answer which of these two components is the more important determinant of obedience in any general sense, as it may not comprehensively manipulate the range of properties that might capture the components or manipulate them in equally powerful ways.

Nevertheless, our analysis indicates that within the confines of 21 of Milgram's conditions, the two components are fairly similar in strength. As Table 4 shows, properties on the Experimenter side of the Teacher (i.e., Experimenter and Teacher-Experimenter relations) have similar overall predictive power as those on the Learner side (i.e., Learner and Teacher-Learner relations), with a small advantage to the Experimenter side. This general finding implies that any interpretation of the Milgram study that neglects one component or the other – that sees the study exclusively through the lens of the Experimenter's influence on the Teacher or the Teacher's disengagement from the Learner, for example – must be incomplete.

One limitation of our analysis is that by focusing on objective properties of the experimental situation it neglects the participant's interpretation of that situation and their understanding of the significance of their behavior. The ambiguity of the situation and apparent skepticism about the experimental set up among many participants [7] all raise questions about how ‘obedience’ – and variations in it across conditions – should be understood within the Milgram paradigm. For example, Milgram's own notes suggest that some conditions were difficult for participants to take seriously. Their degree of belief or disbelief, unmeasured in our analysis, may well have altered the meaning and extent of their ‘obedient’ responding. A second, unavoidable limitation of our analysis is that it could not capture some objective properties of the experimental situation. As Gibson [20] and Perry [7] have shown, the experimenter frequently did not adhere to the published details of the study protocol. Tape recordings show, for example, that he often went beyond the standard ‘four prods’ in ways that are likely to have influenced the delivery of shocks by participants.

Although it is over five decades old the Milgram study is of more than historical significance. Although its meanings remain elusive and continue to generate disagreement, stimulated by new theoretical perspectives and by revelations of methodological weaknesses, attempts to clarify what the study teaches us continue to be important. Whether or not it illuminates the influences on obedience in any general sense, we believe that our analysis helps to extract and systematize some of the patterns within Milgram's complex set of findings. These patterns may help to guide and constrain future interpretations of his study.
